# Trends in poisoning hospitalization and mortality in Taiwan, 1999-2008: a retrospective analysis

**DOI:** 10.1186/1471-2458-11-703

**Published:** 2011-09-16

**Authors:** Wu-Chien Chien, Jin-Ding Lin, Ching-Huang Lai, Chi-Hsiang Chung, Yu-Chen Hung

**Affiliations:** 1 School of Public Health, National Defense Medical Center, No. 161, Section 6, Min-Chuan E. Rd., Neihu, Taipei, 11490, Taiwan; 2 Graduate Institute of Life Sciences, National Defense Medical Center, No. 161, Section 6, Min-Chuan E. Rd., Neihu, Taipei, 11490, Taiwan

**Keywords:** poisoning, mortality, hospitalization, trends

## Abstract

**Background:**

Subjects with non-fatal poisoning may be left with permanent, disabling sequelae, and the resultant long-term use of medical services smay be a burden on the public health care system. The objective of this study was to describe the epidemiology of poisoning in Taiwan from 1999 to 2008.

**Methods:**

We analyzed poisoning-related data of mortality rates sourced from official Taiwanese vital statistics and of hospitalization from the National Health Insurance (NHI) Research Database. The data were age-adjusted to the year 2000 Standard Population to determine 10-year hospitalization and mortality rate trends, which we stratified according to gender, age, and poisoning agent. Poisson regression was used to investigate the trends.

**Results:**

There were 20,260 deaths and 210,021 hospitalizations related to poisoning, with mortality and hospitalization rates of 8.21 per 100,000 and 86.30 per 100,000 population, respectively. Males exhibited higher rates of mortality and hospitalization as a result of poisoning, with the highest risk in those aged 65 years or older. Medicinal drugs followed by pesticides were the two most common agents of poisoning. There was an increasing trend of both poisoning-related mortality and hospitalization rates during the study period, with a greater increase occurring in the hospitalization rate than in the mortality rate.

**Conclusions:**

We found the males aged 65 years or older were at highest risk of poisoning, with medicinal drugs being the leading cause. Hospitalization rates increased more than mortality rates over the 10-year period. Appropriate poisoning prevention programs need to be developed. We should strengthen case management and improve access to health services to increase survival in cases of poisoning.

## Background

Poisoning is a global public health concern. The World Health Organization (WHO) indicated that in 2000, unintentional poisoning resulted in 315,000 deaths worldwide. This represents 6% of all deaths from unintentional injuries, a proportion that is comparable with deaths from unintentional falls [1]. According to statistics from the American Association of Poison Control Centers in 2004, one poisoning case occurred every 13 seconds in the United States (US), with an incidence of 5.5-18.1 per 1,000 population. Altogether there was a yearly total of 200,000 cases of suicide poisoning along with two million cases of unintentional poisoning [2].

Among all unintentional injuries in the US, unintentional poisoning ranks as the second leading cause of death, surpassed only by automobile injuries [3]. According to the US Centers for Disease Control and Prevention, poisoning accounted for the largest increase in mortality rate among all unintentional injuries from 1999 to 2004, with an overall increase of 55.7%, and a 62.5% increase in unintentional poisoning, a 10.8% increase in suicide poisoning, and a 26.6% increase in unspecified poisoning [4]. In addition, according to health statistics in the United Kingdom, poisoning was the second leading cause of death among all unintentional injuries in England and Wales in 2004, ranked only behind fall injuries. As mortality from suicide poisoning declined between 1979 and 2004, mortality from unintentional and drug abuse poisoning rose to become the primary cause of poisoning [5].

According to the Taiwan National Poison Center, the incidence of poisoning in Taiwan is about 0.16-0.22 per 1,000 population [6]. Statistics from the Department of Health showed that a total of 324 Taiwanese subjects died from unintentional poisoning in 2007. The poisoning mortality rate ranks fourth among unintentional injuries, of which the top three causes are automobile injuries, unintentional falls, and drowning [7]. In Taiwan, there was a decreasing trend in poisoning mortality from 1975 to 1998, with a drop of 41.2%. While unintentional and intentional poisoning mortality fell by 40.1% and 55.6%, respectively, there was a 32.5-fold increase in the mortality from unspecified poisoning [8]. In addition, a study showed that between 1986 and 2005, suicides using solid or liquid substances and suicides using gas and vapor were the second and third leading agents of suicide deaths [9].

Most previous studies conducted in Taiwan that focused on poisoning were cross-sectional and failed to present the overall epidemiological characteristics and long-term trends in poisoning. Therefore, this study aimed to describe the epidemiological characteristics and trends of poisoning in Taiwan using a combination of cause-of-death statistics and the National Health Insurance (NHI) Research Database containing hospitalization data.

## Methods

### Data source

The mortality data in Taiwan from 1999-2008 were obtained from the cause-of-death statistics provided by the Department of Health, Executive Yuan [7]. Hospitalization data were extracted from the NHI Research Database [10]. Since its launch on March 1, 1995, the NHI has reached a coverage rate of 99% of hospitals in Taiwan. The NHI Research Database consists of data on both outpatient (including emergency treatment) and inpatient care. Medical institutions are required by law to make claims for outpatient costs and inpatient fees to the NHI Bureau. Hence, the NHI Research Database is an essential resource of representative and evidence-based data for studies in medical and health research, which may play a valuable role in future health policy [10].

Our research required secondary data analysis, and the identification of patients had already been encrypted in the official death statistics and the NHI Research Database in Taiwan; therefore, our research complied with the Helsinki Declaration and protected the privacy of patients.

### Data analysis

We calculated the mortality and hospitalization rates using the mid-year population in the "population by age report" provided by the Ministry of the Interior as the denominator. Data were age-adjusted by the direct method to the 2000 World Standard population obtained from the WHO. Afterwards, we calculated the 10-year trends in poisoning mortality and hospitalization rates and stratified them by gender, age, and causative agent.

The Department of Health age classification, which uses 6 groupings, was adopted as follows: infants and toddlers, aged 0-4 years; children, aged 5-14 years; young adults, aged 15-24 years; mature adults, aged 25-44 years; middle-aged adults, aged 45-64 years; and the elderly, aged 65 years or older. The official death statistics and NHI database employ the ICD-9-CM coding system in Taiwan, so we selected study cases by ICD-9-CM external causes of injury codes (E-Codes), which include "unintentional poisoning, such as by medicinal drugs (E850-E858), alcohol (E860), cleaning solutions (E861), petroleum products (E862), pesticides (E863), corrosives (E864), foodstuffs (E865), other solid and liquid substances (E866), gas distributed by pipeline (E867), carbon monoxide (E868), other gases and vapors (E869), venomous animals and plants (E905), and medicinal drugs causing adverse effects (E930-E948)"; "intentional poisoning, such as suicide using solid or liquid substances (E950), suicide using gases in domestic use (E951), suicide using other gases and vapors (E952), and assault using poisoning (E962)"; and "substances, undetermined whether accidently or purposely inflicted (E980), by unspecified gases in domestic use (E981), and by other unspecified gases (E982)."

For statistical analysis, the independent sample Student t-test, one-way analysis of variance, and Scheffe's *post hoc *comparisons were conducted using SPSS 18.0 software to examine the differences in poisoning mortality and hospitalization rates according to gender, age, and poisoning agent. Poisson regression was used to identify 10-year trends.

## Results

### Epidemiological characteristics

Between 1999 and 2008, there were a total of 20,620 deaths from poisoning in Taiwan, representing a mortality rate of 8.21 per 100,000 population. When considering intent, the number of deaths caused by intentional poisoning was the greatest, with 14,440 deaths (70.03% of all poisoning deaths), and demonstrated the highest mortality rate (5.73 per 100,000 population). The second and third greatest causes of poisoning deaths were unintentional poisoning (17.41%; 1.45/100,000) and unspecified poisoning (12.56%; 1.03/100,000). Men demonstrated a mortality rate 2.3 times higher than women (11.46 *versus *4.92 per 100,000 population). Cross-analysis of gender and intention showed that men had a higher proportion of unintentional poisoning among all poisoning deaths than women (18.71% *versus *14.29%), whereas women had higher proportions of intentional and unspecified poisoning among all poisoning deaths than men (72.77% *versus *68.88% and 12.94% *versus *12.41%, respectively). Cross-analysis of age and intention showed that death from unintentional poisoning was significantly higher in the 25-44 year and 65 years or older age groups (2.59 and 2.18 per 100,000, respectively) compared with other age groups; on the other hand, mortality rates from intentional poisoning were significantly higher in the 65 years or older, 45-64, and 25-44 year age groups (10.63, 9.44, and 8.86 per 100,000, respectively) compared with other age groups (Table 1).

**Table 1:** Poisoning mortality rates by gender and age in Taiwan in 1999-2008 (per 100,000 population)

Types	Age	① 0-4 yrs		② 5-14 yrs		③ 15-24 yrs		④ 25-44 yrs		⑤ 45-64 yrs		⑥ ≧ 65 yrs		Total	
		
	Gender	N	Rate	N	Rate	N	Rate	N	Rate	N	Rate	N	Rate	N	Rate
Unintentional Poisoning ***	Total	38	0.29	56	0.18	329	0.89	1,939	2.59	766	1.56	460	2.18	3,589	1.45
④,⑥ > ⑤ > ③,①,②	Male	15	0.22	35	0.21	215	1.13	1,562	4.12	622	2.54	268	2.55	2,718	2.16
	Female	23	0.36	21	0.14	114	0.63	377	1.02	144	0.58	192	1.83	871	0.73
Drugs ***	Total	4	0.03	1	< 0.01	181	0.48	1,129	1.52	181	0.35	70	0.33	1,566	0.63
(E850-E858)	Male	1	0.02	1	0.01	121	0.63	953	2.53	155	0.62	38	0.36	1,269	0.99
④ > ③,⑤,⑥ > ①,②	Female	3	0.05	0	0	60	0.33	176	0.48	26	0.10	32	0.31	297	0.25
Other utility gas and CO **	Total	26	0.20	48	0.15	87	0.24	295	0.39	134	0.27	56	0.27	646	0.27
(E868)	Male	9	0.13	32	0.19	46	0.25	196	0.52	99	0.40	36	0.33	418	0.34
④ > ⑤,⑥,③,①,②	Female	17	0.27	16	0.11	41	0.23	99	0.27	35	0.14	20	0.20	228	0.20
Pesticide ***	Total	1	0.01	0	0	27	0.08	164	0.22	206	0.44	196	0.93	594	0.24
(E863)	Male	1	0.01	0	0	24	0.13	134	0.35	170	0.74	128	1.21	457	0.37
⑥ > ⑤,④,③,①,②	Female	0	0	0	0	3	0.02	30	0.08	36	0.15	68	0.65	137	0.11
Intentional poisoning ***	Total	25	0.19	29	0.09	840	2.24	6,708	8.86	4,578	9.44	2,260	10.63	14,440	5.73
⑥,⑤,④ > ③,①,②	Male	11	0.16	10	0.06	567	2.94	4,829	12.59	3,279	13.59	1,308	12.23	10,004	7.88
	Female	14	0.22	19	0.13	273	1.51	1,879	5.03	1,299	5.34	952	9.01	4,436	3.56
Not explicitly ***	Total	11	0.09	14	0.04	158	0.42	1,244	1.65	703	1.44	461	2.18	2,591	1.03
⑥ > ④,⑤ > ③,①,②	Male	8	0.12	6	0.04	98	0.51	939	2.45	507	2.09	244	2.27	1,802	1.42
	Female	3	0.05	8	0.05	60	0.33	305	0.82	196	0.80	217	2.08	789	0.64
Total ***	Total	74	0.57	99	0.31	1,327	3.55	9,891	13.10	6,047	12.43	3,155	14.98	20,620	8.21
⑥,④,⑤ > ③ > ①,②	Male	34	0.50	51	0.31	880	4.57	7,330	19.16	4,408	18.21	1,809	17.04	14,524	11.46
	Female	40	0.63	48	0.32	447	2.47	2,561	6.88	1,639	6.71	1,346	12.96	6,096	4.92

The data show only the top 3 agents of unintentional poisoning

ANOVA (*p < .05; **p < .01; ***p < 0.001), Scheffe's *post hoc *comparisons

A cross-analysis of gender and intention revealed that the proportion of unintentional poisoning in hospitalized male patients was higher than in their female counterparts (76.42% *versus *69.11%), while the proportions of intentional and unspecified poisoning were higher in hospitalized female patients than in their male counterparts (21.24% *versus *15.19% and 9.64% *versus *8.39%, respectively). With regard to hospitalization rate, men demonstrated a 1.04 times higher rate of hospitalization for poisoning than women (88.03 *versus *84.78 per 100,000). A cross-analysis of age and intention showed that the 65 years or older and 45-64 year age groups had significantly higher rates of hospitalization for unintentional poisoning (291.58 and 90.87 per 100,000, respectively), and subjects aged 65 years or older demonstrated the highest rate of hospitalization for intentional poisoning (27.26 per 100,000) (Table 2).

**Table 2:** Poisoning hospitalization rates by gender and age in Taiwan in 1999-2008 (per 100,000 people)

Types	Age	① 0-4 yrs		② 5-14 yrs		③ 15-24 yrs		④ 25-44 yrs		⑤ 45-64 yrs		⑥ ≧ 65 yrs		Total	
		
	Gender	N	Rate	N	Rate	N	Rate	N	Rate	N	Rate	N	Rate	N	Rate
Unintentional Poisoning***	Total	5,455	42.50	3,733	11.90	9,026	24.67	30,047	39.52	42,670	90.87	62,114	291.58	153,045	63.31
⑥ > ⑤ > ①,④,③,②	Male	3,034	45.28	2,152	13.19	4,696	24.94	16,427	42.59	23,479	101.20	32,724	301.99	82,512	67.61
	Female	2,421	39.46	1,581	10.51	4,330	24.39	13,620	36.36	19,191	80.81	29,390	281.02	70,533	58.98
Drugs ***	Total	1,943	14.86	398	1.27	2,360	6.40	6,303	8.40	4,363	9.19	7,563	35.39	22,930	9.77
(E850-E858)	Male	1,006	14.70	220	1.35	951	5.00	2,714	7.15	2,047	8.79	3,580	32.87	10,518	8.88
⑥ > ① > ⑤,④,③ > ②	Female	937	15.04	178	1.18	1,409	7.88	3,589	9.69	2,316	9.61	3,983	37.91	12,412	10.68
Animals and plants ***	Total	179	1.39	353	1.12	634	1.73	2,757	3.61	3,479	7.31	1,03	9.15	9,305	3.85
(E905)	Male	111	1.64	237	1.44	508	2.69	2,137	5.53	2,352	9.93	1,54	10.97	6,499	5.32
⑥ > ⑤ > ④ > ③,①,②	Female	68	1.11	116	0.77	126	0.71	620	1.64	1,127	4.73	749	7.31	2,806	2.36
Pesticide ***	Total	200	1.34	53	0.17	241	0.68	1,588	2.08	2527	5.44	1,709	8.25	6,328	2.62
(E863)	Male	131	1.63	33	0.20	188	0.99	1,186	3.08	1878	8.24	1,249	12.01	4,666	3.85
⑥ > ⑤ > ④ > ①,③,②	Female	69	1.02	20	0.13	63	0.35	402	1.06	648	2.71	460	4.45	1,662	1.38
Intentional poisoning ***	Total	66	0.51	204	0.64	3,249	16.89	17,114	22.78	8,681	17.79	5,770	27.26	38,084	15.39
⑥,④,⑤ > ③,②,①	Male	44	0.65	65	0.39	2,549	13.35	6,753	17.72	4,137	17.31	2,856	26.58	16,404	13.14
	Female	22	0.36	139	0.90	3,700	20.64	10,361	27.98	4,544	18.30	2,914	28.04	21,680	17.74
Not explicitly ***	Total	278	2.17	215	0.68	1,862	5.06	5,723	7.59	4,455	9.33	6,359	29.82	18,892	7.66
⑥ > ④,⑤ > ③,①,②	Male	160	2.40	100	0.61	805	4.25	2,636	6.89	2,236	9.52	3,116	28.79	9,053	7.28
	Female	118	1.91	115	0.76	1,057	5.92	3,087	8.30	2,219	9.16	3,243	30.89	9,839	8.06
Total ***	Total	5,799	45.18	4,152	13.22	17,137	46.62	52,884	69.89	55,806	117.99	74,243	348.67	210,021	86.36
⑥,④,⑤ > ③ > ①,②	Male	3,238	48.33	2,317	14.19	8,050	42.54	25,816	67.21	29,852	128.03	38,696	357.35	107,969	88.03
	Female	2,561	41.74	1,835	12.17	9,087	50.95	27,068	72.65	25,954	108.27	35,547	339.96	102,052	84.78

The data show only the top 3 agents of unintentional poisoning

ANOVA (*p < .05; **p < .01; ***p < 0.001), Scheffe's *post hoc *comparisons

### Long-term trends

**Figure 1:**
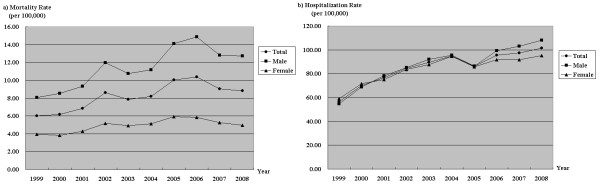
**Long-term trends in poisoning mortality and hospitalization rates by gender**.

Between 1999 and 2008, there was an increasing trend in overall poisoning mortality rate with a significant 47.2% increase (*p *= .002). Men demonstrated a larger increase in mortality rate than women during this time (57.6% *versus *26.2%, *p *= .001 and .012, respectively) (Figure 1a). There was also a rising trend in overall poisoning hospitalization rate, with a 79.1% increase, *p *< .001. Men had a larger increase in hospitalization rate than women (98.1% *versus *61.4%, *p *< .001 and *p *= .001, respectively) (Figure 1b).

**Figure 2:**
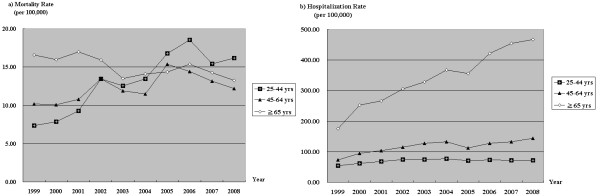
**Long-term trends in poisoning mortality and hospitalization rates by age**.

The population aged 65 years or older demonstrated a declining trend in mortality with a drop of 19.9% over the 10-year period (*p *< .001). However, the 25-44 year age group showed a rising trend in mortality rate, with a 120.7% increase (*p *< .001), and in 2005, the rate in this group surpassed that of subjects aged 65 years or older to become the primary high-risk group for poisoning (Figure 2a). Considering hospitalization, the age group of 65 years or older demonstrated the largest rise in hospitalization rate, with an increase of 165.7% (*p *< .001), followed by the 45-64 and 25-44 year age groups, with increases of 96.1% and 30.8%, respectively (*p *< .001 and .055, respectively) (Figure 2b).

Mortality from unintentional poisoning declined by 35.8% between 1999 and 2008 (*p *< .001). While mortality from intentional poisoning showed an increase of 120.0% (*p *= .001), mortality from unspecified poisoning decreased by 42.7%, but it failed to reach statistical significance (*p *= .396) (data not shown). There was a 123.1% rise in the unintentional poisoning hospitalization rate (*p *< .001), which was in line with the overall hospitalization rate. While the intentional poisoning hospitalization rate decreased by 23.3% (*p *= .151), the unspecified poisoning hospitalization rate increased by 54.1% (*p *< .001) (data not shown).

**Figure 3:**
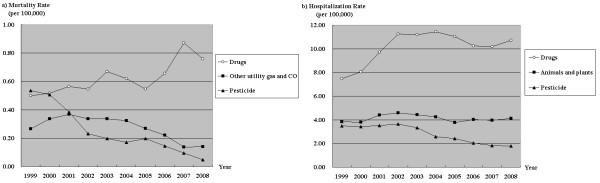
**Unintentional poisoning mortality and hospitalization rates of top 3 poisoning agents**.

After analyzing the causes of unintentional poisoning, we found that the primary agent of unintentional poisoning was medicinal drugs, followed by carbon monoxide, and pesticides. While most causes of unintentional poisoning demonstrated a decreasing trend in mortality rate, unintentional poisoning by medicinal drugs showed a 51.9% increase (*p *< .001) (Figure 3a). Unintentional poisoning by medicinal drugs constituted the leading cause of hospitalization due to unintentional poisoning, followed by venomous animals and plants, and pesticides. In consideration of trends in hospitalization rates, different methods of unintentional poisoning showed varying 10-year trends (Figure 3b).

## Discussion

### Poisoning mortality and hospitalization by gender

Our analysis of overall poisoning mortality revealed that men had higher poisoning mortality than women, regardless of intention; this finding is consistent with the 2000 WHO report that indicated a higher global mortality from unintentional poisoning in men [1]. As for hospitalization, we found that men had a higher rate of hospitalization resulting from unintentional poisoning, and women had a higher rate of hospitalization resulting from intentional poisoning; our findings differed somewhat from the previous literature. In the US, both the 2001-2003 health statistics from Colorado [11], and a study on emergency care data from 2001-2004 [12] indicated a higher poisoning hospitalization rate in women than in men, regardless of intention.

### Poisoning mortality and hospitalization by age

We found that older adults aged 65 years or older showed the highest poisoning mortality among all age groups, regardless of intention. Corresponding to our finding, the 2000 WHO report also demonstrated that subjects aged 80 years or older worldwide had the highest mortality rate from unintentional poisoning, followed by subjects in the 45-59 and 60-69 year age groups [1]. On the other hand, our study was similar to previous research in a hospital center in Taiwan, which showed that the mortality risk in subjects aged 60 and older was 4.3 times more than in those aged under 60 [13]. However, some studies found different trends from ours. In China, a hospital-based study found that the highest rates of poisoning mortality were in adults aged 30-39 years [14]. In south India, a study of poisoning suicide showed that youths aged 20-29 had the highest mortality rate [15]. Regarding poisoning mortality trends, our study found that the population aged 65 years or older demonstrated a significantly declining trend in mortality; the results were similar to the European study of Petridou et al. [16].

Older adults aged 65 years or older demonstrated the highest poisoning hospitalization rate among all age groups, regardless of intention; this finding was different from previous literature. The 2001-2003 health statistics in Colorado in the US suggested that adolescents aged 15-19 years were at highest risk of hospitalization for intentional poisoning, with 98.1 cases per 100,000 subjects [11]. On the other hand, an epidemiological study using emergency care data from 2001 to 2004 in Sri Lanka revealed that adolescents aged 15-19 years demonstrated the highest incidence of non-lethal intentional poisoning (248 per 100,000 subjects), a rate that decreased with age [17]. However, our study was similar to previous research that showed the elderly aged 65 or older had the highest medicinal drug poisoning hospitalization rate [18]. The phenomenon may be related to the fact that this population is less well educated and has a lower literacy rate, resulting in a lack of knowledge and skills related to handling items safely. In addition, the existence of multiple illnesses, multiple drug medication and poor physical conditions may also increase the risk of hospitalization or mortality as a result of poisoning.

### Poisoning mortality and hospitalization by intention

The majority of unintentional poisoning deaths was caused by medicinal drugs, followed by carbon monoxide, and pesticides. The most frequent cause of unintentional poisoning hospitalization was medicinal drugs, followed by venomous animals and plants, and pesticides. A number of previous studies showed similar results that medicinal drugs [11,19-21] and pesticides [13-15] were the primary causes of poisoning-related mortality and hospitalization. This phenomenon may result from the widespread prescribing and easy availability of medicinal drugs and pesticides, compared with other poisoning methods. Therefore, the following discussion will focus on the long-term trends in poisoning from medicinal drugs and pesticides.

### Trends in mortality and hospitalization from poisoning by medicinal drugs

This study revealed a rising trend in mortality and hospitalization rates from unintentional poisoning caused by medicinal drugs. This result was similar to a previous study in Taiwan showing an increase in unintentional drug poisoning mortality rate of 51.9% [18]. Since the launch of the NHI in Taiwan in 1995, the general public has had better access to prescription drugs, such as sleeping pills, sedatives, and analgesics, which may increase the possibility of drug misuse and increase mortality resulting from unintentional drug poisoning [20]. The promulgation of the Drug Harvard Relief Act in Taiwan in 2000 allows timely relief, without resorting to lawsuits or pleads, to victims of unforeseeable adverse reactions from proper use of legal drugs that results in death, disability, or serious illness [21]. Therefore, the launch of the Act may have encouraged reporting of drug poisoning deaths and thus could have increased the death count from unintentional drug poisoning.

### Trends in mortality and hospitalization rates from poisoning by pesticides

This study revealed decreasing trends between 1999 and 2008 in mortality and hospitalization rates from unintentional poisoning by pesticides. In Korea from 1996 to 2005, mortality from pesticides rose for both genders [22]. In Central America, there was also an increase in both incidence and mortality rates of pesticide poisoning from 1992 to 2000, which might be related to increased pesticide imports during the same period of time [23]. In Thailand from 1987 to 1996, both injuries and deaths from pesticide poisoning decreased; however, the numbers might be an underestimation because most locals failed to seek medicinal help in the event of poisoning [24]. In Japan from 1991 to 1996, poisoning by pesticides was on the decline, which could result from the decrease in paraquat poisoning [25].

As a result of social transformations in Taiwan in recent years, the area of farmland has been decreasing. The traditional agriculture-centered Taiwanese society depends more and more on business, traditional industry, and high-technology industry, and organic farming has also gradually replaced traditional farming, resulting in a large decrease in pesticide use. Between 1951 and 1992, the proportion of agricultural workers in the Taiwanese population decreased from 56% to 12%, while that of industrial workers rose from 16% to 40% [26]. According to the 2009 statistics from the Council of Agriculture, Executive Yuan, there has been a decreasing trend in the amount of people employed in the agricultural industry, from approximately 710,000 people in 2002 to about 540,000 people in June, 2009 [27]; this trajectory has coincided with the increasing importance attached to environmental protection and food safety issues by the public. Since 1972, a total of 119 agricultural chemicals have been banned due to high toxicity, teratogenicity after long-term exposure, tumorigenicity, and/or environmental pollution [28]. The abovementioned reasons may well account for the decline in pesticide poisoning mortality rate.

### Limitations

The variables analyzed in this study were limited by the cause-of-death statistics in Taiwan and the hospitalization data retrieved from the NHI Research Database. Certain information was not available, such as clinical biochemistry data, education level of subjects, smoking habits, and use of alcohol.

Our study was limited with regards to cause-of-death statistics; when registering the cause of death, the Department of Health only records the first three digits of the E-Codes. The absence of the fourth digit hindered us from extracting additional detailed information. For example, we were unsuccessful in differentiating between organic phosphorus pesticides and organic chlorine pesticides in agricultural chemical poisoning cases and were unable to compare the mortality data against the hospitalization data.

After an injury, posttraumatic care can be categorized into no medical care or self-care, outpatient care, emergency care, inpatient care, and death. However, because the outpatient and emergency treatment data provided by the NHI Research Database failed to include E-Codes, we were unable to classify those patients receiving such care by the poisoning method based on the ICD-9-CM system. As a result, only deaths and hospitalized patients for poisoning injuries were analyzed in this study, while those receiving other types of care were excluded.

## Conclusions

Males demonstrated higher rates of poisoning mortality and hospitalization than females in Taiwan between 1999 and 2008. The most affected age group was 65 years or older. The main agents causing death were medicinal drugs, carbon monoxide and pesticides, while the main agents resulting in hospitalization were medicinal drugs, venomous animals/plants, and pesticides. Hospitalization rates increased more than mortality rates over the 10 years.

The results indicated that more attention should be paid to prevention of poisoning in the public health system in Taiwan. In addition, we should adopt the suggestions of Bose et al. [29] to strengthen case management and access to health services to improve survival in cases of poisoning.

## Competing interests

The authors declare that they have no competing interests.

## Authors' contributions

WCC contributed to the study design, obtained the data and drafted the paper. JDL and CHL provided suggestions for revision of the manuscript. CHC contributed to analyze the data. YCH contributed to interpretation of the data. All authors have read and approved the final manuscript.

## Pre-publication history

The pre-publication history for this paper can be accessed here:

http://www.biomedcentral.com/1471-2458/11/703/prepub
